# Effectiveness of Yi-Zhi-An-Shen granules on cognition and sleep quality in older adults with amnestic mild cognitive impairment: protocol for a randomized, double-blind, placebo-controlled trial

**DOI:** 10.1186/s13063-019-3607-x

**Published:** 2019-08-20

**Authors:** Shengnan Yue, Ting He, Baiyang Li, Yanqin Qu, Hongmei Peng, Jinxin Chen, Ming Lei, Chongli Chen, Wenbin Wu

**Affiliations:** 1 School of Clinical Medicine, Chengdu University of Traditional Chinese Medicine, No. 1177 Liu-tai Avenue, Chengdu, 611137 Sichuan Province People’s Republic of China; 2 Department of Geriatrics, Hospital of Chengdu University of Traditional Chinese Medicine, No. 39 Shi-er-qiao Road, Chengdu, 610072 Sichuan Province People’s Republic of China

**Keywords:** Amnestic mild cognitive impairment, Sleep, Gut microbiome, Older adults

## Abstract

**Background:**

Amnestic mild cognitive impairment (aMCI) is a syndrome characterized by significant forgetfulness that does not meet the criteria of dementia. Individuals with aMCI are at greater risk of progressing to dementia. Current studies suggest that good sleep quality is linked with preserved cognition in the elderly, and sleep complaints are common among the elderly with amnesia. Therefore, improving their sleep may be helpful for maintaining and improving their cognitive capacity. According to the theory of traditional Chinese medicine, Yi-Zhi-An-Shen is an herbal compound which may ameliorate forgetfulness and sleep disorders. As growing evidence indicates that the gut microbiome is associated with major mental symptoms, a hypothesis was proposed that Yi-Zhi-An-Shen granules (YZASG) might work by alternating microbial abundance and diversity. In this study, the investigators intend to assess the efficacy of YZASG on global cognition in the elderly suffering from aMCI and evaluate its safety as well as its potential mechanisms via sleep quality, fecal microbial 16S ribosomal DNA and metagenomics analyses, and serum markers.

**Methods/design:**

This study is a randomized, double-blind, placebo-controlled clinical trial. A total of 80 patients (aged 60–85 years) will be recruited and allocated randomly to a treatment group and a placebo group in a 1:1 ratio and will then be administered YZASG or isodose placebo three times a day. The intervention course is 16 weeks, with an 18 months follow-up. The primary outcome is the Alzheimer’s Disease Assessment Scale-Cognitive Subscale. Secondary outcome measures are the Mini-Mental State Examination, Montreal Cognitive Assessment, Pittsburgh Sleep Quality Index, serum concentrations of immunological factors and inflammatory cytokines, and fecal microbiota. Fecal microbiota will only be collected at the baseline and endpoint of the intervention.

**Discussion:**

The results of this trial will be conducive to assessing the safety and effectiveness on cognition of YZASG in intervening aMCI among the elderly and determining if it takes effect via the improvement of sleep quality, regulation of gut microbiota, and concentration of certain serum markers.

**Trial registration:**

ClinicalTrials.gov, NCT03601000. Registered on 26 July 2018.

**Electronic supplementary material:**

The online version of this article (10.1186/s13063-019-3607-x) contains supplementary material, which is available to authorized users.

## Background

Alzheimer’s disease (AD), one common type of dementia worldwide, is a neurodegenerative disease characterized by insidious onset and progressive cognitive decline [1]. It not only detrimentally affects physical condition and quality of life in patients, but it also places a huge burden on both the family and society [2]. The prevalence of AD is 3.21% reportedly among the old in China [3]. It was proposed recently that research strategies for AD should focus on the preclinical stages — preclinical AD and mild cognitive impairment (MCI) due to AD — which are relatively ideal for intervention [4].

Patients with MCI experience cognitive decline which is more severe than expected for an individual’s age and education level but which does not obviously affect daily function [5]. Also, the elderly with MCI are at a high risk of developing dementia [6]. Nearly 90% of older adults with amnestic MCI (aMCI), i.e., MCI with memory complaints and the main subtype of MCI, reportedly progress to AD and share similar pathophysiological characteristics with AD patients [7]. Nowadays, standard clinical management of MCI includes managing its risk factors, as there are still only limited pharmaceutical options for treating MCI, according to the National Institute on Aging-Alzheimer’s Association (NIA-AA) working group guidelines [8]. Thus, effective drugs and other interventions are expected to be discovered to reduce the rate of progression from MCI to dementia.

Brain areas and systems of neurotransmitters involving regulation of the sleep-wake cycle relate primarily to memory and cognition [9–11]. Individuals with various types of cognitive impairment generally have sleep disturbance [9, 12–14], and one study also suggested that the severity of these sleep disorders closely relates to that of cognitive decline [14]. Moreover, older adults with bad sleep quality always suffer poor cognitive performance [15, 16], which might be related to higher levels of AD-associated amyloid-β in the brain [17]. Structural imaging data indicated that insomnia is also associated with a decreased volume of brain tissue, including the hippocampus [18]. A disorder of the circadian rhythm often accompanies a high risk of cognitive decline and negatively affects cognition in multiple ways. Therefore, a sleeping problem is one risk factor of impaired cognitive function.

Several studies suggested that besides improving the cognitive performance and ability of daily living in individuals with AD, acetylcholinesterase inhibitors (ChEIs), like donepezil, galantamine, and rivastigmine, also improved the mental behavioral symptoms and sleep quality of these patients [19–21]. Furthermore, some trials indicated that melatonin could improve cognitive symptoms in patients with MCI and might delay their conversion to dementia [22, 23]. As sleep disturbance appears to be linked with both aging and cognitive decline, strengthening the sleep quality of the elderly might be an effective therapeutic target to slow the deterioration or improve cognitive impairment.

According to recent studies on MCI or AD which involved donepezil [7], galantamine [24], memantine [25], and solanezumab [26], pharmaceutical interventions showed poor amelioration of cognitive deficits. It might be that the single pharmacological target of these drugs did not adequately address the specific multiple pathophysiological characteristics. However, traditional Chinese medicine (TCM) is known for its multitargeted approach and could be used to address the complicated pathophysiological changes.

In the light of TCM theory, kidney deficiency is the basis of amnesia and other cognitive deficits, while phlegm and blood stasis are significant pathological factors. For this reason, experiments and trials have been carried out and have shown specific effects against cognitive decline, involving, e.g., *Alpiniae oxyphyllae fructus* [27], *Ligusticum wallichii* [28–30], *Curcuma* [31, 32], *Fructus gardeniae* [33, 34], and *Radix notoginseng* [35, 36]. Meanwhile, an herbal formula named Yi-Zhi-An-Shen granules (YZASG) has been developed, composed of *Lophatherum gracile* and the medical herbs mentioned above, which is designed for aiding the treatment of cognitive deficits. Table 1 details the pharmacological targets of the components in YZASG, which could be involved in the management of MCI. There have been preclinical research studies contributing to the standardization of YZASG [37] and the mechanisms of its potential actions in animal experiments [38]. Some indications have also manifested its safety and efficacy for improving cognitive function in clinical practice without causing daytime dysfunction.

**Table 1:**
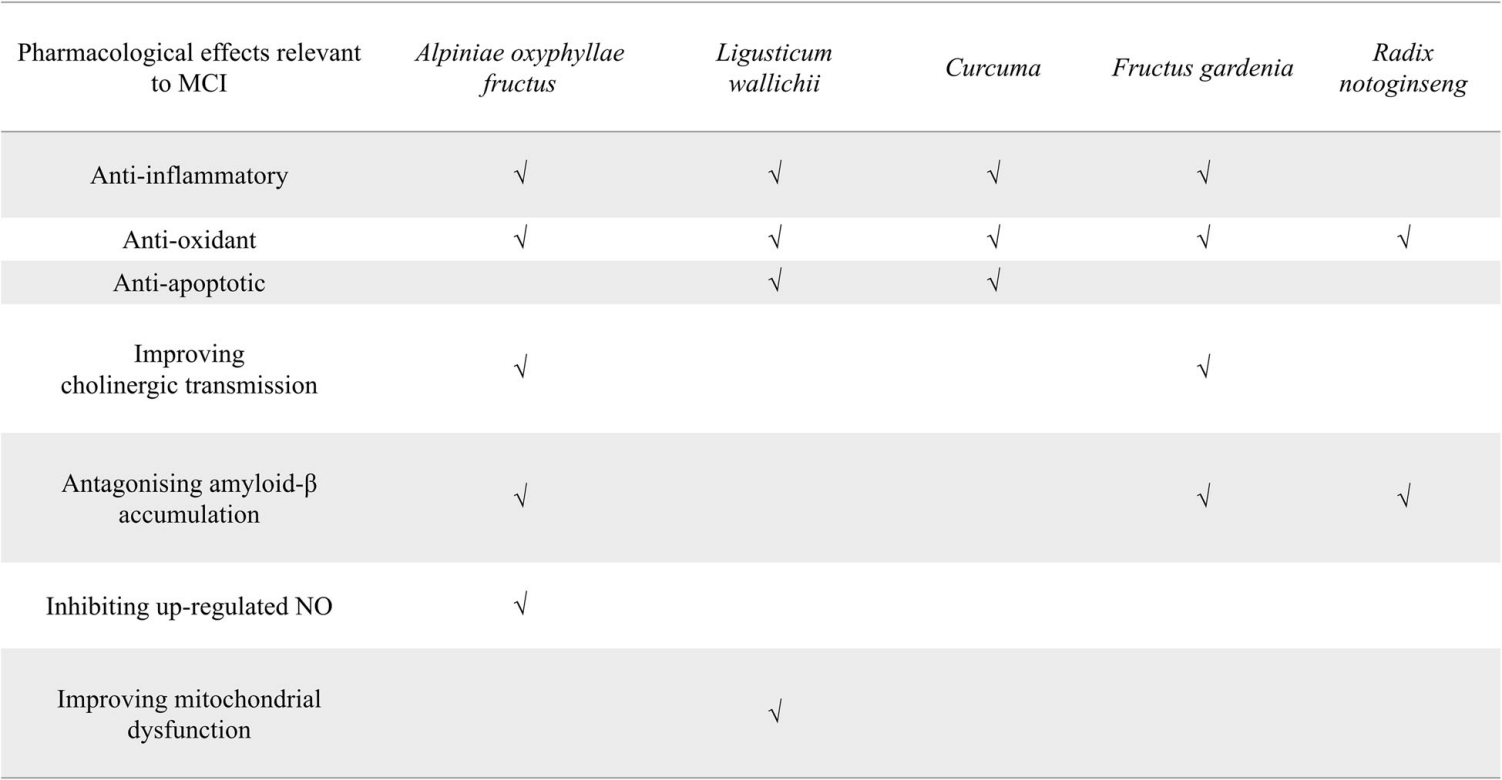
Multitarget mechanisms underlying pharmacological effects of YZASG components

Based on the theory of TCM, the actions of YZASG include soothing the nerves, that is, making a person calm down and helping him/her sleep. Besides, according to the emerging hypothesis of the brain-gut axis, numerous studies have shown that changes of the gut microbiome and its metabolites exert an influence on cognition impairment [39–41]. Because it is administered orally, we note that the mechanism of action of YZASG might be via the gut microbiota. Consequently, a clinical trial with rigorous design is needed to confirm its safety in old individuals with MCI and its efficacy on cognitive performance, and also to explore its potential mechanisms.

## Methods

### Objectives

The primary objective of this study is to evaluate the efficacy of YZASG in optimizing cognitive performance over time in elderly individuals with aMCI. Secondly, the investigators intend to assess whether YZASG can improve sleep quality among aMCI patients, and this herbal formula’s safety will also be assessed. Finally, participants’ serum samples and fecal genomic DNA will be extracted to analyze the differences of the indices of metabolism, cellular immune function, and gut microbiota between old individuals with aMCI and those with normal cognition.

### Design

This study is a randomized, double-blind, placebo-controlled trial with a 16-week intervention and an 18 months follow-up assessment. The current protocol (version v1.1) meets the principles of the Declaration of Helsinki, is in accordance with Standard Protocol Items: Recommendations for Interventional Trials (SPIRIT) guidelines (see Additional file 1), and was approved by the Medical Ethics Committee of Hospital of Chengdu University of TCM. Signed informed consent forms will be obtained from participants, and during the consent process, the caregiver or informant of the potential participant should be present. The participants will be recruited from the communities, outpatient clinics of Hospital of Chengdu University of TCM, and the Welfare Institution of Emei Civil in Sichuan Province, China. The Medical Ethics Committee has thoroughly reviewed this study, and the ethical approval covers all these study sites.

**Fig. 1:**
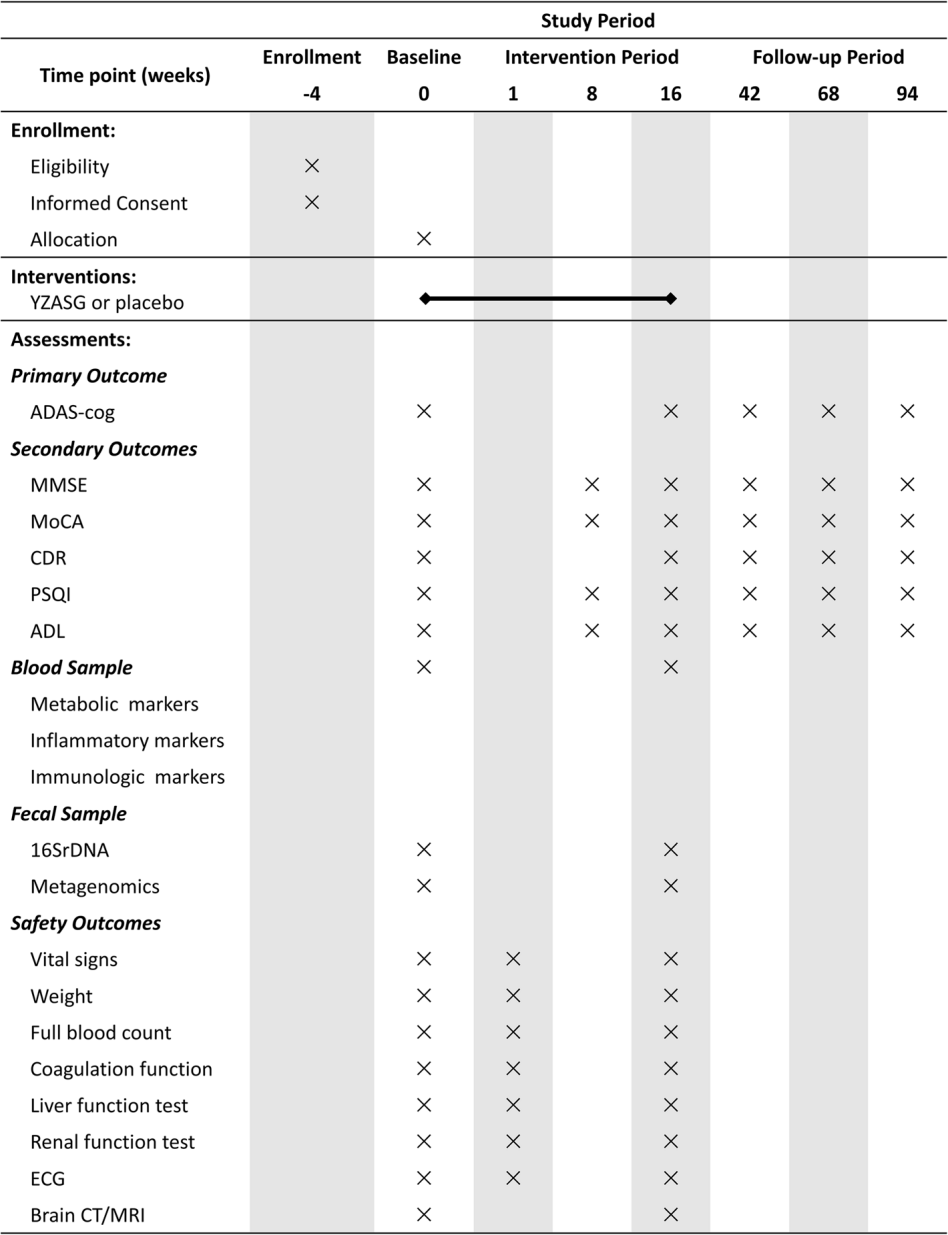
Schedule of interventions and assessments. *Abbreviations*: *ADAS-cog* Alzheimer’s Disease Assessment Scale-Cognitive Subscale, *MMSE* Mini-Mental State Examination, *MoCA* Montreal Cognitive Assessment, *CDR* Clinical Dementia Rating, *PSQI* Pittsburgh Sleep Quality Index, *ADL* activities of daily living, *16S rDNA* 16S ribosomal deoxyribonucleic acid, *ECG* electrocardiography, *CT* computed tomography, *MRI* magnetic resonance imaging. Vital signs include an individual’s temperature, breaths and pulse per minute, and blood pressure

The enrolled participants will be randomly assigned into the YZASG group or the placebo group with an allocation ratio of 1:1 using a statistical package. Allocation was concealed using batch numbers generated with SAS 9.2 software (SAS Institute, Cary, NC, USA) by a statistician. A unique code will be assigned to each newly enrolled participant and preserved by the trial management board. The statistician expert who acts as the coder will be shielded from subject recruitment and the statistical analysis, which will be performed by another statistician independent of the study group. Both participants and the research team are blinded to allocation. There will be six measurement sessions during this study, including the intervention period and the 18 months period (Fig. 1). Any changes to the study protocol will be communicated to the study investigative team and the approving ethics committee.

### Participants

The investigators anticipate recruiting 80 participants from Hospital of Chengdu University of TCM, communities, and the Welfare Institution of Emei Civil Administration. All patients will undergo a standard medical examination and neuropsychological testing to ensure the correct diagnosis of aMCI.

The inclusion criteria are:

1. Assigned informed consent from the subject to participate in the study and continued willing consent for participation
2. Age from 60 to 85 years with a diagnosis of aMCI
3. Educational level of at least 6 years
4. Availability of a caregiver or informant who can assist in completing rating scales for the duration of the study
5. Cognitive complaints reported by the subject and confirmed by the caregiver or informant
6. Clinical Dementia Rating (CDR) global score of 0.5 and memory item score of 0.5
7. Mini-Mental State Examination (MMSE) score of 24–30 (for participants with educational level of 6 years, MMSE score of 20–30)
8. Diagnostic and Statistical Manual of Mental Disorders, Version 5 (DSM-V) criteria of dementia not fulfilled.

A patient will be excluded if he or she:

1. Has been previously enrolled in this study and received the investigational product
2. Has received an investigational product within 30 days prior to screening
3. Has received disease-modifying therapy in the past 6 months (e.g., donepezil, rivastigmine, galantamine, memantine, or any other existing drugs that are declared to have the function of improving cognition)
4. Has a known allergy to the study drug or any of its constituents
5. Has a history of alcohol abuse or alcohol dependency in the 3 years prior to study entry, or is an alcoholic or drug addict, as determined by the investigator
6. Has ongoing clinically significant (as judged by the investigator), metabolic, or any other disease that could currently cause impaired memory (e.g., untreated thyroid disease, vitamin or other nutritional deficiencies, chronic kidney or liver disease)
7. Has memory impairments that can be attributed to a disease or condition other than an early-phase neurodegenerative syndrome
8. Has a parkinsonian movement disorder
9. Uses psychoactive medications that would affect the subject’s ability to reliably perform neurocognitive testing or create uncertainty in distinguishing between the effects of the psychoactive medication and the subject’s underlying cognitive impairment (e.g., benzodiazepines, sedatives, antipsychotics)
10. Has a history of major recurrent depressive disorder (per DSM-V) within the last 5 years prior to screening
11. Has a brain tumor or other intracranial lesion, a disturbance of cerebral spinal fluid circulation (e.g., normal pressure hydrocephalus), and/or a significant history of head trauma or brain surgery
12. Has signs of major cerebrovascular disease, with a modified Hachinski Ischemia Score (mHIS) of more than 4, or as verified by medical history and/or brain magnetic resonance imaging (MRI) or computed tomography (CT) scan
13. Has severe visual or hearing impairments and cannot cooperate with examinations
14. Has a severe digestive system disease
15. Has received antibiotics within 60 days prior to screening.

Reasons for participant withdrawal and dropout are as follows (1) voluntarily withdrawal, (2) loss of follow-up, (3) poor compliance and presence of severe adverse effects, (4) revealing and uncovering blind in urgency, (5) misdiagnosis, (6) using forbidden drugs or treatments in the course of the trial, (7) taking no medication during the trial, (8) no evaluable records after medication. Reasons for withdrawing participants will be recorded in case report forms (CRFs), and the last of their data will be included in the data analysis. All these criteria will be ascertained by the supervisor of this study.

### Interventions

**Fig. 2:**
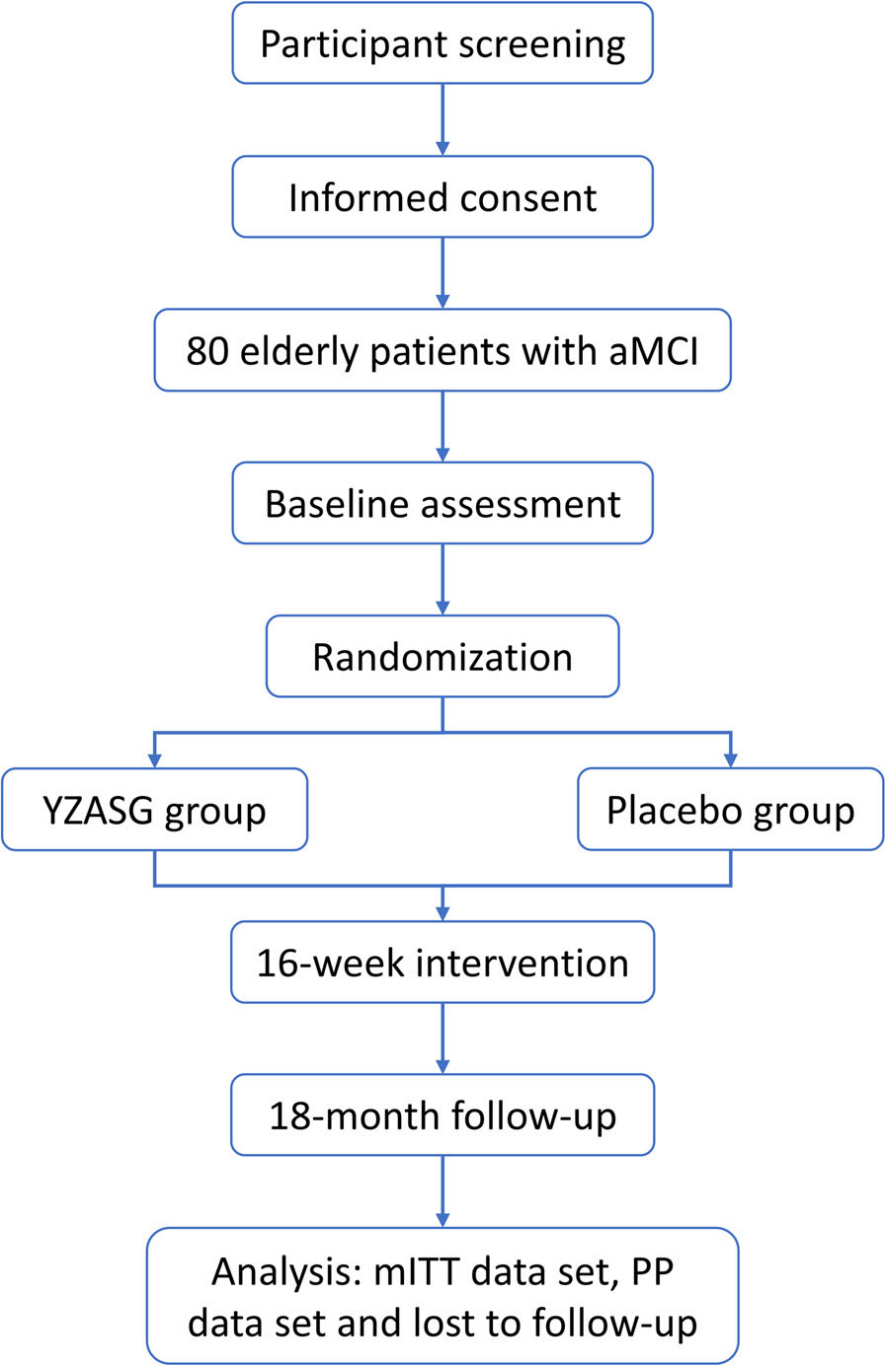
Flowchart of the trial

A flowchart of this trial is presented in Fig. 2. All the participants will receive the same basic treatment, including health education, moderate aerobic exercise (30–60 min per day), and general nutritional support. The participants assigned to the YZASG group will take the Yi-Zhi-An-Shen granule, which is composed of YiZhiRen (*Alpinia oxyphylla* Miq.) 5 g, SanQi (*Panax notoginseng*) 3 g, ChuanXiong (*Ligusticum chuanxiong* Hort.) 10 g, ZhiZi (*Gardenia jasminoides* Ellis) 10 g, YuJin (*Curcuma longa* L.) 10 g, and DanZhuYe (*Lophatherum gracile*) 10 g, while those in the placebo group will take a placebo made from starch which has the same shape, color, smell, taste, texture, package, and lot number as the intervention. Participants will be instructed to dissolve the granules into 100 ml of boiled water and to take the solution orally at a temperature between 30 and 37 °C three times daily for 16 weeks. Each granule is prepared by Sichuan Neo-Green Pharmaceutical Technology Development Co., Ltd., Sichuan, China, according to the standards of Good Manufacturing Practice (GMP). During the entire study period, relevant health care and treatment for medical needs will be permitted as long as there are no adverse reactions with YZASG and no hindering of the study process.

The actual dose taken by one patient within the range of 80 to 120% of the recommended dose is considered eligible for the protocol plan. The package, drug name, function and indication, usage and dosage, storage conditions, validity period, and name of the manufacturer will be marked, and a tag indicating ”trial use” will be attached. Drugs must be kept at the appropriate temperature in a dry, cool, and shady place. Drug administrators should take back unused drugs to estimate participant compliance and record this in the CRFs.

### Outcomes

#### Primary outcome

Cognitive decline is measured using the Chinese version of the Alzheimer’s Disease Assessment Scale-Cognitive Subscale (ADAS-cog11). This scale was also chosen to calculate the sample size, with a 4-point change in score as the measure of clinical significance [42]. The total possible score is 70; a higher score indicates greater severity of impairment. The specific hypothesis is that the increase from baseline to endpoint will be significantly less (by at least 2.5 points) than that for the placebo, which will be considered effective in this study.

The ADAS-cog11 will be assessed at baseline (before intervention), at 16 weeks (the end of the intervention), and at 6, 12, and 18 months after the intervention.

#### Secondary outcomes

Secondary measures include the MMSE, Montreal Cognitive Assessment (MoCA, Changsha version), CDR, Pittsburgh Sleep Quality Index (PSQI), and activities of daily living (ADL) scores, the gut microbiome, and serum markers.

The MMSE is an 11-question measure that tests five areas of cognitive function (orientation, registration, attention and calculation, recall, and language). The maximum score is 30, and a score below 24 is considered abnormal for dementia screening.

The MoCA (Changsha version) will also be used to evaluate general cognitive function, as it contains visuospatial processing and organizational capability parts and can make up for the shortcomings of the MMSE. The total score for the MoCA is 30, with a higher number indicating a more intact cognitive function. The MoCA has been shown to be a promising tool to detect MCI and early AD.

The CDR will be used as an assistant evaluation for patients’ dementia severity. It scores 0–3, with higher scores indicating more severity. It is a semi-structured interview performed with the patient and caregiver (informant), characterizing six domains of cognitive and functional performance. The CDR sum of boxes (CDR-SB), scored 0–18, will also be applied to assess patients’ cognitive status, with higher scores indicating worse functioning.

The PSQI will be used to assess participants’ comprehensive quality of sleep, which involves sleep quality, sleep duration, sleep efficiency, sleep disorders, daytime dysfunction, sleeping aids, etc. The total score for the PSQI is 21, with a higher score indicating a worse sleep quality.

ADL will be assessed including basic activities of daily living (BADL) and instrumental activities of daily living (IADL). An individual’s BADL will be evaluated mainly by the subjects’ performance from the perspectives of bathing, dressing, grooming, initiation, toileting, and feeding, with six items and a sum of scores ranging from 0 (normal) to 24 (complete dependence on others). A modified Lawton IADL scale will be used to measure the IADL of a subject, with eight items and a sum of scores ranging from 0 (normal) to 32 (complete dependence on others).

These clinical tests will be administered by a trained, certified clinician or rater experienced in the assessment of patients with cognitive deficits. The rater who conducts the CDR for a patient cannot complete any other rating scales for the same patient, and will be blinded to the results of all other neuropsychological scales. The previously described scales will be assessed at baseline (before the intervention), at 8 and 16 weeks (during the intervention), and at 6, 12, and 18 months after the intervention.

Blood samples will be collected from all participants to further assess the mechanisms of YZASG via changes in serum metabolic, inflammatory, and immunologic markers. All these tests will be entrusted to the laboratory medicine departments of Sichuan Academy of Medical Science & Sichuan Provincial People’s Hospital for conduction.

Fecal genomic DNA will be extracted from frozen stools using a QIAamp DNA Mini Stool Kit (Qiagen, Hiden, Germany), obtained from the patients with aMCI and 15–20 participants with normal cognition at baseline and at the end of the intervention. After polymerase chain reaction (PCR) amplification, DNA fragments will be sequenced on an Illumina HiSeq 2500 instrument and an Illumina HiSeq X instrument for 16S ribosomal DNA (16S rDNA) and metagenomics analyses (which will be chosen from some representative samples in the results of the 16S rDNA analysis), respectively, at Biomarker Technologies Co, Ltd. (Beijing, China) to analyze the differences in gut microbiome between patients with aMCI and individuals with normal cognition. When the results are available, the investigators will also assess the changes in gut microbiome between the treatment group and the placebo group after the intervention of YZASG in the same way.

#### Safety outcomes

To assess the safety of YZASG compared to placebo in subjects with aMCI, the investigators will record the incidence and severity of treatment-emergent adverse events (TEAEs) and clinically important changes in safety assessment results. These safety indicators, including vital signs, weight, clinical laboratory tests, physical and neurological exams, electrocardiography (ECG), and CT/MRI scans, will be gathered at the baseline and at the end of the 16th week.

## Statistical considerations and data management

### Sample size

Using pre-intervention and post-intervention scores obtained from Miao et al. [43], on the basis of a non-inferiority trial principle, one-sided test, at α = 0.05, at least 33 patients are needed for inclusion in the treatment group who will be administered YZASG to achieve a prospective power of 90% (i.e., β = 0.1) and detect a minimum clinical between-group difference of 1.30 on ADAS-cog [43] at 16 weeks. Allowing for a maximum dropout rate of 20%, the number of subjects in the treatment group has been set to 40 patients with aMCI. With an allocation ratio of 1:1, 80 subjects are required.

### Statistical analysis

The analysis will be conducted by another statistician, from the National Clinical Trial Center of Chinese Medicine (Chengdu, China), who will also be blinded to the whole trial, using SAS 9.2 software (Cary, NC, USA) and SPSS 21.0 software (IBM, Armonk, NY, USA).

The analysis data set will consist of a modified intention-to-treat data set, a per protocol (PP) set, and a safety data set. All mechanism and efficacy analyses will be conducted according to the modified intention-to-treat (mITT) principle. The mITT data set will include the participants who have completed at least one observation since the intervention began. The PP population will only include participants who adhered to the trial protocol and completed the clinical trial; the minimum compliance rate for participants taking the investigational drugs in the PP data set is 80%. A safety analysis will be conducted according to the safety data set, which will include any participants who were randomly assigned to and took at least one dose of the investigational drug. Missing values will be replaced by the last observation carried forward (LOCF) method. ADAS-cog (including its monomial item) changes from baseline and the secondary outcomes will be assessed using an analysis of covariance with treatment groups as factors and baseline values as covariates. Mean differences will be used to express effect sizes. The baseline homogeneity of the baseline characteristics and differences between the two groups will be analyzed with Fisher’s exact test or the χ^2^ test for categorical measures and with the *t* test or Wilcoxon rank-sum test for continuous measures. The statistical significance is defined as a one-sided *p* value of < 0.05 and 90% confidence interval.

For the gut microbial 16S rDNA analysis, sequenced data will be interpreted using the bioinformatics tools programmed in the Ion Reporter Software. Based on the specified similarity, Quantitative Insights into Microbial Ecology (QIIME) algorithms will be used to classify operational taxonomic units (OTUs) and statistically analyze biological information, then to understand the diversity and abundance of the flora community, and further to determine the bacterial diversity within a sample (α-diversity) and among all the samples (β-diversity). The α-diversity includes four indicators that represent total number, richness, phylogenetic diversity, and dispersible uniformity of species and community abundance. These four algorithms, including binary Jaccard, Bray-Curtis, weighted UniFrac, and unweighted UniFrac, will be performed to analyze the β-diversity to compare the similarity of different samples in species diversity. According to the preceding data, principal component analysis will be conducted to observe the differences between floras. Additionally, multivariate data analysis with principal component analysis on the diversity indices and comparisons of genus and species level data will be performed to reveal differences in the microbial composition between individuals with normal cognition and those with aMCI. Metastats software will be used to perform *t* tests on the species abundance data between the two groups, and a *p* value will be obtained; then by correcting the *p* value, a *q* value will also be obtained. Subsequently, according to the *p* or *q* value, species that cause the differences in the microbial composition of the two sets will be screened out. Significant analyses between two groups will be performed at the level of classification of the gate, class, subject, family, genera, and species, respectively. The SparCC algorithm will be used to conduct correlation analyses (including positive and negative correlation) and statistical tests. Next, a co-expression analysis network map will be drawn using Python. The subsequent statistical analyses will be performed with the R Programming Language 3.0.1 (NZL).

For the metagenomics analysis, after getting Clean Reads, a taxonomic analysis will be conducted to measure species composition and abundance information of samples. After the significance test of differences has been performed, a *p* value will be obtained. Then by correcting the *p* value, the false discovery rate (FDR) will be obtained. The Benjamini-Hochberg FDR adjustment will be used to account for the number of taxa tested in each comparison.

### Data management

All data will be stored on a secure server with two back-up copies on external hard drives. Paper-based forms will be digitized and the original copies stored in locked filing cabinets in the archive of Good Clinical Practice (GCP) in the Department of Geriatrics, and managed by a department staff member who is external to the research team. All participants are de-identified upon randomization and referred to on all forms by a participant ID. A password-protected spreadsheet stored on the secure server links all participant names to ID codes so that re-identification can occur if required. As this is a relatively small investigator-initiated trial, a data monitoring committee and auditing process are not required.

## Quality control and monitoring

Each trial center has a project manager who takes charge of the quality of research. All investigators were qualified and trained before the study began. After the baseline measurement, the 16 weeks of intervention medication will be dispensed. Participants will be required to return any unused medication every 2 weeks in order to determine compliance. The number of returned granules will be counted by a department member who is external to the study team. During the whole course of the trial, attentive follow-up will also be conducted every 2 weeks. Participants who exit the study early will be contacted via telephone and requested to complete the exit interview.

To monitor safety, participants will be referred to Hospital of Chengdu University of TCM and the local cooperating hospital of the Welfare Institution of Emei Civil Administration, which is next to the institution. Standard blood safety tests (full blood count, blood coagulation function, liver and renal function tests) and ECG tests will be carried out at the baseline and at the end of the 1st and 16th weeks of intervention, while brain CT/MRI scans will be done at the baseline and at the end of the 16th week. Participants are also encouraged to share their annual physical checkup reports with the investigators in this study.

Adverse events will be closely monitored and recorded throughout the whole course of the study. If a serious adverse event occurs, if the study drug is suspected to be a potential cause and receiving appropriate medical care is identified to be essential for the participant, then unblinding of that participant will occur, and at least two persons should be present. After being treated, that participant will be discontinued from the subsequent investigation and regarded as a dropout case. The investigators will also visit that participant regularly and record his/her reactions and treatments of the adverse events until the endpoint of this study. This task will be performed by another group of physicians who are independent of the groups of investigators who are in charge of enrolment, administering drugs and evaluating participants. A development update safety report must be submitted annually to the Medical Ethics Committee of Hospital of Chengdu University of TCM.

The random code and allocation information will be kept concealed from the study team and participants until the end of this study when all statistical analyses have been finished.

## Discussion

YZAS has shown effectiveness in improving cognitive performance and neuroinflammation in animal experiments. Provided that it can also be proved to address cognitive deficits in this clinical trial, it may be shown to act via multiple mechanisms involved in the pathology of aMCI, which will be determined in the study.

Assuming that old individuals with aMCI almost always experience poor sleep, this study may provide an evidence-based medical approach to improving sleep quality and then maintaining cognition among these patients. However, in terms of testing specifically the differences of fecal microbiome between the old with normal cognition and those with aMCI, this project has a relatively small sample size, which is calculated according to the primary outcome. Owing to the number and the length of the tasks, the participants’ burden is a bit high; breaks will be scheduled during each period of follow-up assessments. The results of this randomized controlled trial will inform the development of future interventional studies to prevent or delay cognitive decline among patients with MCI or dementia.

### Trial status

This trial (protocol version v1.1, 18 December 2017) began recruitment on 21 April 2018 and is currently ongoing. The recruitment is expected to be completed by the end of 2019.

## Additional file

Additional file 1: SPIRIT 2013 checklist: recommended items to address in a clinical trial protocol and related documents. (DOC 138 kb)

## Data Availability

The data sets used and/or analyzed during this study are available from the corresponding author on reasonable requests.
